# Optimal depolarization sequence for cardiac resynchronization therapy patients

**DOI:** 10.1093/ehjopen/oeag165

**Published:** 2026-10-08

**Authors:** Ksenia Sedova, Jan Azarov, Peter M Van Dam, Karol Curila, Pavel Leinveber, Josef Kautzner

**Affiliations:** Department of Biomedical Technology, Faculty of Biomedical Engineering, Czech Technical University in Prague, Sitna Sq. 3105, Kladno 272 01, Czech Republic; Department of Cardiology, Institute for Clinical and Experimental Medicine, Videnska 1958/9, Prague 140 21, Czech Republic; Department of Biomedical Technology, Faculty of Biomedical Engineering, Czech Technical University in Prague, Sitna Sq. 3105, Kladno 272 01, Czech Republic; Center for Digital Medicine and Robotics, Jagiellonian University Medical College, Kopernika 7E, Krakow 31-034, Poland; Department of Cardiology, Cardiocenter, Third Faculty of Medicine, Charles University, University Hospital Kralovske Vinohrady, Ruska 87, Prague 100 00, Czech Republic; International Clinical Research Center, St. Anne’s University Hospital, Pekarska 53, Brno 602 00, Czech Republic; Department of Cardiology, Institute for Clinical and Experimental Medicine, Videnska 1958/9, Prague 140 21, Czech Republic

**Keywords:** Cardiac dyssynchrony, Cardiac resynchronization therapy, Heart Failure, Depolarization gradients

## Abstract

**Aims:**

Electrical ventricular dyssynchrony is a hallmark of intraventricular conduction disease in heart failure with reduced ejection fraction. Although cardiac resynchronization therapy (CRT) is effective, a proportion of patients with electrical dyssynchrony evidenced by an electrocardiogram (ECG) remain non-responders. Besides the QRS duration, visualization and quantification of the depolarization sequence could contribute to improving CRT response. The study aimed to evaluate the CineECG-derived mean temporo-spatial isochrone trajectory of depolarization in CRT patients.

**Methods and results:**

Among the adult cohort of 121 patients who underwent CRT implantation, there were 93 (33% female) responders and 28 (7% female) non-responders. ECGs recorded before and immediately after CRT implantation were collected and analysed using the CineECG modality with a generic heart/torso model. The CineECG-derived path directions were computed for the QRS on anteroposterior, interventricular, and apicobasal axes within the cardiac anatomy. Spatial depolarization characteristics were tested for the association with left ventricle reverse-remodelling at 6-month follow-up. The pre-implantation depolarization sequence was predominantly from the right to the left ventricle, and after the CRT implant, it has been transformed into an apicobasal direction. In multivariate logistic regression, the pre-implant QRS duration, apicobasal depolarization gradient, and CRT-induced QRS shortening independently predicted CRT response. The Akaike information criterion indicated that combining all three yielded the strongest predictive model.

**Conclusion:**

CineECG-derived depolarization gradients, which reflect ventricular activation sequence, changed significantly after CRT, and the pre-implantation apicobasal gradient independently predicted echocardiographic outcome, with a less pronounced gradient associated with better haemodynamic response.

## Graphical Abstract

**Graphical Abstract:**
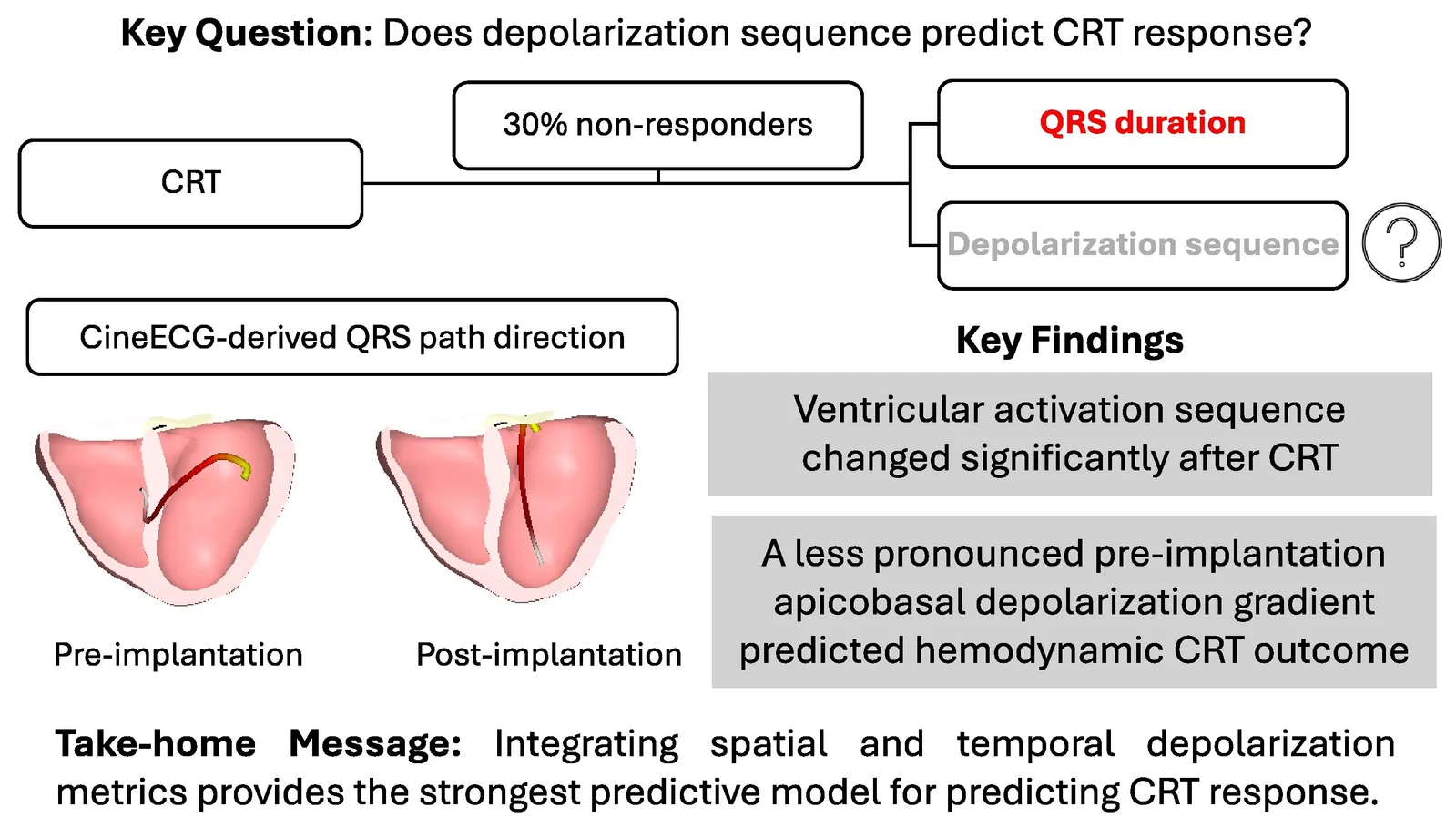

## Introduction

Cardiac resynchronization therapy (CRT) is the therapy of choice in heart failure patients with reduced ejection fraction and intraventricular conduction abnormalities. Since CRT aims to correct these abnormalities and improve haemodynamics, the electrocardiogram (ECG) is a primary tool for assessing the type of electrophysiological abnormality. Current guidelines for CRT patient selection are based mostly on QRS duration.^1^ However, using these non-specific criteria may result in a higher proportion of non-responders to CRT.^1,2^ Therefore, there is an unmet need to develop a better strategy.

Non-response to CRT may have different reasons. The patient may be in advanced heart failure, limiting any palpable improvement in haemodynamic status. Alternatively, the current ECG criteria may not be sufficient to estimate ventricular dyssynchrony. Despite attempts to identify morphological criteria of the true left bundle branch block (LBBB) as an indicator of ventricular dyssynchrony,^3–5^ the absence of a reliable quantitative description of spatial (or spatiotemporal) parameters of intraventricular conduction limits the use of such metrics in clinical practice.

Recently proposed CineECG, a cardiac anatomy-based ECG modality, has the capability to quantify the spatial characteristics of ventricular depolarization for better visualization of myocardial electrophysiological processes.^6–8^ This study aimed to evaluate the CineECG-derived mean spatiotemporal QRS path directions of depolarization before and after CRT implantation and test these metrics for their association with echocardiographic CRT outcomes.

## Methods

### Study design

This retrospective observational study involved patients who underwent CRT implant at the St. Anne’s University Hospital, Brno, Czech Republic. The study group comprised 121 patients with heart failure and reduced left ventricular ejection fraction (LVEF), who had a clinical indication for CRT based on current ECG criteria. For each patient, the standard 12-lead ECG obtained in sinus rhythm before CRT implant (pre-implantation ECG) and immediately after device implantation (post-implantation ECG) were exported from the Ultra-High-Frequency Electrocardiograph (UHF-ECG; ISI Brno, Cardion, FNUSA, Czech Republic)^9^ for further analysis. Precordial leads were positioned according to the joint AHA/ACC/ESC recommendations, while limb leads followed the Mason–Likar configuration. All ECG recordings were performed by trained clinical staff, with careful attention to consistent electrode placement to ensure high-quality and reproducible ECG acquisition. ECGs were analysed automatically using the CineECG software, which provided quantitative measurements of QRS duration and depolarization gradients. ECG analyses were subsequently reviewed by a blinded observer to ensure data accuracy. Clinical response to CRT was assessed 6 months after CRT implantation and defined as a reduction in left ventricular end-systolic volume of ≥10%, as measured by transthoracic echocardiography (i.e. reverse remodelling). All data analysed were anonymous, and the study was performed according to the Declaration of Helsinki and was approved by the institutional ethics committee.

### ECG data analysis

Electrocardiographic data were processed using the CineECG method, where the generic heart/torso model of a 57-year-old male with average body build was used to construct the average electrical pathway within the cardiac anatomy from the 12-lead ECG.^6,7^ The position of the sequence of depolarization is constructed following the vectorial electrical gradients (vectorcardiogram/VCG) derived from the standard 12-lead ECG, which means that areas where the VCG direction points from areas that depolarize earlier to areas that depolarize later.^8^ The VCG signal is computed by computing the lead vectors and the ECG amplitude for every unipolar lead (VR, VL, VF, and V1–V6). These lead vectors are determined by standard electrode positions on a thorax model of a 57-year-old male. The spatial summation of every ECG sample of the nine unipolar signals results in the VCG. Based on this VCG, the CineECG is computed, i.e. the average location of ventricular electrical activity throughout the cardiac cycle. This effectively represents a moving dipole estimation approach. To compute the direction of the electrical gradient for the QRS interval, the position at the end of the interval is subtracted from the position at the start of the interval. This 3D-vector is then normalized, resulting in a standardized length of 1. The 3D-gradient vector thus ranges between −1 and 1, expressed in the local heart coordinate system, representing the QRS pathway directions computed in the posterior-anterior, right-to-left, and apex-to-base direction. Additionally, the CineECG automatically computed a median QRS waveform with an automatically estimated QRS duration from which the depolarization gradients are computed.

### Statistics

ECG parameters were compared between two groups of patients referred to as responders or non-responders using the Mann–Whitney U test for continuous variables and χ² or Fisher’s exact test for categorical variables. Pre-implantation and post-implantation ECG measures were compared with the Wilcoxon paired test. The association between spatial parameters of depolarization and QRS duration was tested in a multivariate linear regression analysis.

The association between ECG (and/or CineECG) parameters of ventricular depolarization and the haemodynamic outcome of CRT was tested in logistic regression analysis. After that, different subsets of potential predictors were compared according to the Akaike information criterion (AIC): AIC = −2 ln(*L*) + 2*k*, where *L* is the likelihood of the fitted model and *k* is the number of estimated parameters (including the intercept), with minimal AIC indicating the best model. For all tests, the difference was considered significant at *P* < 0.05.

## Results

### Patient characteristics

The study population included 121 patients with reduced ejection fraction and LBBB or non-specific intraventricular conduction delay. Clinical characteristics are summarized in *Table 1*. The mean age of the studied population was 66 ± 10 years, and 27% were females. The patients had dilated as well as ischaemic cardiomyopathy with a mean QRS duration of 148 ± 19 ms and LVEF 24 ± 7% before CRT implant. All patients were in sinus rhythm, and LBBB pattern corresponding to Strauss criteria was present in 84% of the cohort and ischaemic aetiology in 40%.

**Table 1:** Patient clinical characteristics (*n* = 121)

Age, years	66 ± 10
Female	33 (27%)
Body mass index, kg/m^2^	29 ± 5
Delated cardiomyopathy	80 (66%)
Coronary artery disease	49 (40%)
LBBB Strauss	102 (84%)
Atrial hypertension	82 (68%)
Diabetes mellitus	34 (28%)
Beta-blockers	106 (88%)
Statins	70 (58%)
Diuretics	108 (89%)
LV ejection fraction, %	24 ± 7%
Native QRS duration, ms	148 ± 19

Values are mean ± SD or number (%) for continuous and categorical variables, respectively.

LBBB, left bundle branch block; LV, left ventricle.

As expected, before CRT implantation, patients presented with a wide QRS complex and predominantly right-to-left depolarization gradient (*Table 2*) due to delayed activation in the left ventricle. Posterior-anterior and apicobasal gradients were less pronounced and indicated directions of depolarization from posterior to anterior and from the apex to base (*Table 2*). Despite differences in cardiomyopathy aetiology, depolarization gradients did not differ significantly between patients with ischaemic and dilated cardiomyopathy (*P* > 0.05). In multivariate linear regression analysis with backward elimination, QRS duration was significantly associated with posterior-anterior depolarization gradient (B −14.59, 95% CI −25.44 to −3.74, *P* = 0.009). The negative sign of the regression coefficient indicates that longer QRS duration was associated with a posterior-to-anterior rather than an anterior-to-posterior direction of depolarization sequence.

**Table 2:** Medians and interquartile ranges (IQR) of ventricular depolarization characteristics before and after CRT implantation

Parameter	Pre-implantation	Post-implantation	Change	*P* value
QRS duration, ms	147 [IQR 137–160]	118 [IQR 103–132]	30 [IQR 8–46]	<0.001
QRS path direction AP	−0.16 [IQR −0.32–0.05]	0.00 [IQR −0.23–0.31]	−0.12 [IQR −0.45–0.17]	0.002
QRS path direction RL	0.86 [IQR 0.73–0.93]	0.15 [IQR −0.20–0.43]	0.62 [IQR 0.37–0.97]	<0.001
QRS path direction AB	0.35 [IQR 0.08–0.60]	0.86 [IQR 0.60–0.96]	−0.41 [IQR −0.78 to −0.13]	<0.001

Values are median (interquartile range).

AB, apicobasal; AP, posterior-anterior; RL, right-left.

Cardiac resynchronization therapy resulted in a shortening of the QRS duration and significant changes in all depolarization gradients as compared to the baseline (*Table 2*). The right-to-left gradient decreased, the posterior-anterior gradient decreased and nearly disappeared, while the apicobasal gradient increased (*Table 2*). These changes together suggest that the predominant depolarization sequence during CRT is apicobasal. In multivariate linear regression analysis with backward elimination, QRS duration after CRT implant was significantly associated with apicobasal (B 14.33, 95% CI 5.39–23.26, *P* = 0.002) and posterior-anterior depolarization gradient (B −8.92, 95% CI −17.51 to −0.33, *P* = 0.042). The signs of the regression coefficients indicate that longer QRS duration was associated with apex-to-base and posterior-to-anterior depolarization sequences.

Responders and non-responders to CRT treatment differed from each other in all tested pre-implantation depolarization characteristics (*Table 3*). As compared to the non-responders, the responders demonstrated longer QRS duration, more pronounced posterior to anterior and right to left depolarization gradients, and a less pronounced apex to base depolarization gradient (*Figure 1*). In contrast, neither of the post-implantation parameters differentiated responders from non-responders, except for the magnitude of QRS duration shortening, which was greater in responders (*Table 3*).

**Figure 1:**
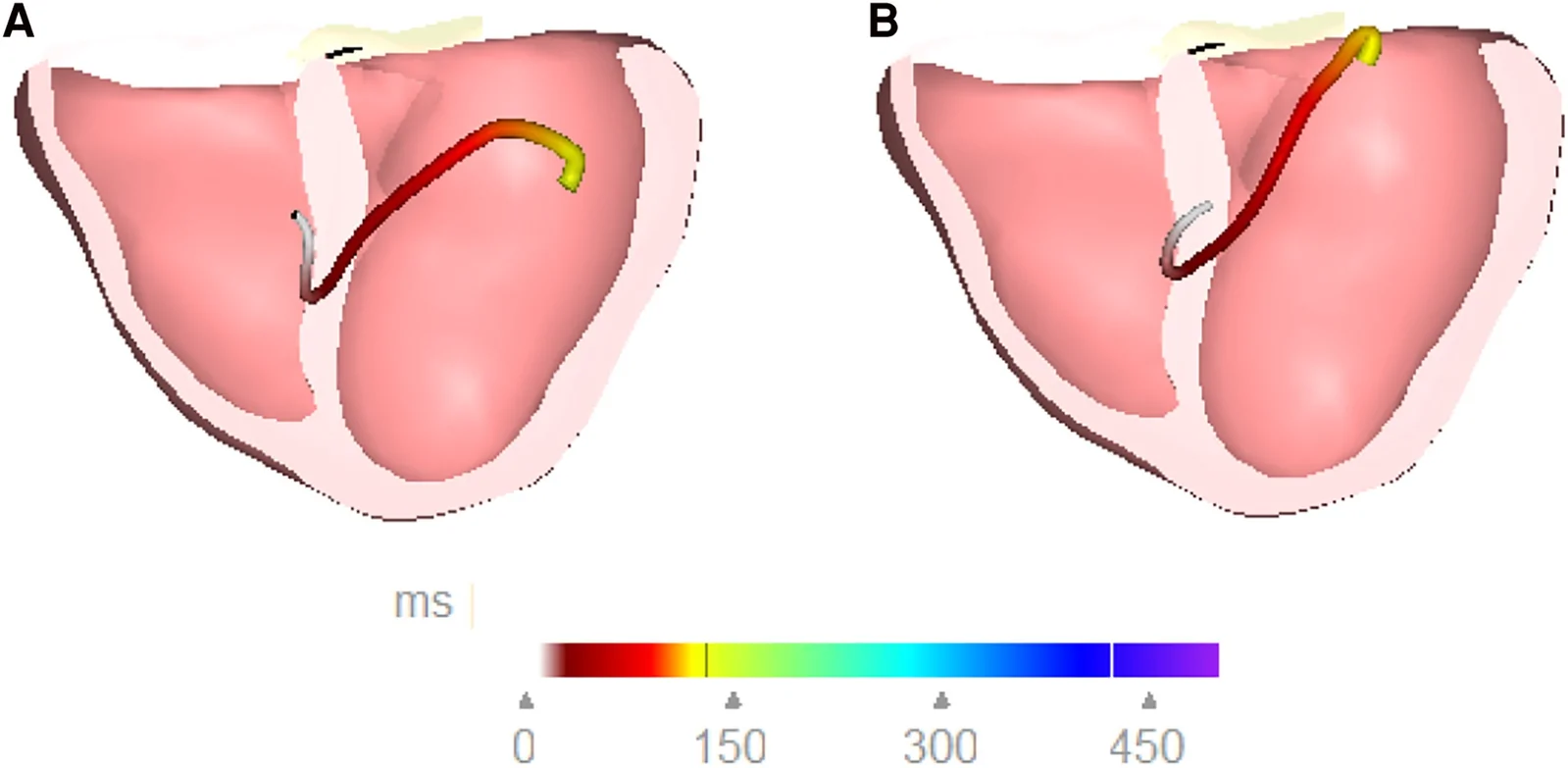
Pre-implantation sequence of depolarization in responder (*A*) and non-responder (*B*) to cardiac resynchronization therapy. (*A*) Responder demonstrating a less pronounced apicobasal QRS path direction (0.09). (*B*) Non-responder demonstrating a more pronounced apicobasal QRS path direction (0.67).

**Table 3:** Comparisons of median ventricular depolarization characteristics between responders and non-responders to CRT treatment

Parameter	Responders	Non-responders	*P* value
Pre-implantation ECG			
QRS duration, ms	151 [IQR 140; 163]	138 [IQR 123; 146]	<0.0001
QRS path direction AP	−0.18 [IQR −0.32; 0.01]	0.03 [IQR −0.26; 0.31]	0.025
QRS path direction RL	0.88 [IQR 0.77; 0.94]	0.75 [IQR 0.49; 0.87]	0.003
QRS path direction AB	0.28 [IQR 0.01; 0.56]	0.52 [IQR 0.32; 0.70]	0.009
Post-implantation ECG			
QRS duration, ms	118 [IQR 102; 132]	118 [IQR 111; 138]	0.455
QRS path direction AP	−0.01 [IQR −0.21; 0.30]	0.05 [IQR −0.24; 0.44]	0.633
QRS path direction RL	0.15 [IQR −0.18; 0.47]	0.18 [IQR −0.24; 0.41]	0.794
QRS path direction AB	0.86 [IQR 0.57; 0.96]	0.85 [IQR 0.64; 0.94]	0.891
Change			
QRS duration, ms	33 [IQR 20–49]	10 [IQR 1–24]	<0.001
QRS path direction AP	−0.20 [IQR −0.45–0.15]	0.00 [IQR −0.37–0.25]	0.246
QRS path direction RL	0.67 [IQR 0.37–0.99]	0.55 [IQR −0.33–0.90]	0.259
QRS path direction AB	−0.44 [−0.83 to −0.14]	−0.32 [IQR −0.49–0.07]	0.109

Values are median (interquartile range).

AB, apicobasal; AP, posterior-anterior; RL, right-left; ECG, electrocardiogram.

The parameters of ventricular activation that were different in responders as compared with non-responders to CRT were further tested for the association with CRT response in multivariate logistic regression analysis (*Table 4*). The longer pre-implantation QRS duration, the less pronounced apicobasal pre-implantation depolarization gradient (*Figure 1*), and the greater magnitude of QRS shortening by CRT appeared to be independent predictors of CRT response. To address the potential influence of clinical characteristics, the identified ECG predictors of CRT response were further tested in a multivariable logistic regression analysis adjusted for age, sex, and ischaemic cardiomyopathy. After adjustment, pre-implantation QRS duration (OR = 1.052, 95% CI 1.008–1.097, *P* = 0021), apicobasal depolarization gradient (OR = 0.067, 95% CI 0.011–0.413, *P* = 0.004), and QRS shortening after CRT (OR = 1.041, 95% CI 1.011–1.073, *P* = 0.008) remained independently associated with CRT response. To further evaluate the relative performance of different predictor combinations, multivariable models including different subsets of ECG parameters were compared using AIC. Among different subsets of predictors, the model including these three parameters (pre-implantation QRS duration, its change by CRT, and the pre-implantation apicobasal depolarization gradient) was the best according to AIC analysis (*Table 5*).

**Table 4:** Multivariate logistic regression analysis of CRT response predictors (enter method)

Variable	OR (95% CI)	*P* value
Pre-implantation QRS duration	1.059 (1.017–1.103)	0.005
Pre-implantation QRS path direction AP	0.570 (0.093–3.495)	0.543
Pre-implantation QRS path direction RL	0.641 (0.031–13.167)	0.773
Pre-implantation QRS path direction AB	0.096 (0.012–0.749)	0.025
QRS duration change	1.028 (1.002–1.056)	0.036

AB, apicobasal; AP, posterior-anterior; RL, right-left; OR, odds ratio; CI, confidential interval.

**Table 5:** Comparison of different predicting models according to Akaike information criterion (AIC)

Included parameters	*k*	−2ln(*L*)	AIC
Pre-implantation QRS duration	2	109.4	113.4
Pre-implantation QRS duration and QRS duration change	3	103.5	109.5
Pre-implantation QRS duration and pre-implantation QRS path direction AB	3	99.9	105.9
Pre-implantation QRS duration and QRS path direction AB, QRS duration change	4	94.3	102.3
Pre-implantation QRS duration and all pre-implantation QRS path directions	5	99.4	109.4

AB, apicobasal; AIC, Akaike information criterion; *k*, number of tested parameters, including the intercept; *L*, likelihood of the fitted model (from multivariate logistic regression analysis).

## Discussion

This study employed the CineECG to better predict the clinical response to CRT. We tested the ventricular depolarization gradients quantitatively measured as QRS path directions on three anatomical axes of the heart as predictors of the long-term CRT outcome. We demonstrated that the apicobasal depolarization gradient prior to treatment was independently associated with the response to CRT at the 6-month follow-up.

The main goal of CRT is to improve cardiac dyssynchrony, which requires reproducible measurement of dyssynchrony and description of its extent and/or type that could be effectively treated by CRT. According to current guidelines,^1^ dyssynchrony is assessed by QRS duration, which provides the temporal metric of it. However, the notoriously high proportion of non-responders in such populations^1,2^ suggests that patient selection for CRT should be improved. Development of spatial in addition to (or instead of) temporal criteria of dyssynchrony seems to be a reasonable option.

A number of previous studies^10–12^ suggested that the spatial characteristics of ventricular activation should be considered independently of temporal parameters in order to predict the CRT response. However, these considerations mostly addressed the activation pattern after the onset of CRT therapy. Probably, the most prevalent approach to assess pre-implantation spatial dyssynchrony is to detect a true LBBB pattern on ECG.^3–5^ As compared to other conduction abnormalities, the presence of a true LBBB pattern indicates a more probable response to CRT.^13–15^ However, the LBBB and non-LBBB spatial patterns are assessed using morphological criteria, i.e. the presence or absence of qualitative ECG signs,^3,5,16,17^ and therefore, their quantification might be difficult.

The advantage of the CineECG is that it provides a clear visualization and quantification of the depolarization and repolarization sequences. It has been successfully used in clinical contexts where spatial characteristics of cardiac activity are important.^18–21^ One example is a prediction of acute haemodynamic response in CRT patients.^21^ In the present study, we used this approach with the assumption that the conduction disorders manageable by CRT are characterized not only as a prolonged duration of ventricular activation (estimated as QRS duration) but also by an abnormal depolarization sequence (estimated as QRS path directions). We tested the hypothesis that the spatial abnormalities in the ventricular depolarization pattern contribute independently to ventricular dyssynchrony and affect the outcome of CRT.

Indeed, we found that the pre-implantation apicobasal QRS path direction was independently inversely associated with the CRT response at 6-month follow-up. Inclusion of the apicobasal gradient (but not all gradients) into the regression model enhances its predictive value (*Table 5*). It implies that a typical CRT responder should have a base-to-apex depolarization sequence or at least should not have a pronounced apex-to-base depolarization sequence. The spread of activation from the base to the apex probably represents the most abnormal activation pattern and is likely to be corrected by CRT. On the other hand, we did not observe any associations between CRT response and post-implantation depolarization metrics, except for the magnitude of temporal resynchronization (QRS shortening). This finding is supported by recent meta-analysis, demonstrating the association of the QRS duration shortening after CRT implantation with clinical and echocardiographic response.^22^ Our findings emphasize the importance of depolarization gradients assessed prior to CRT implantation for predicting CRT response, rather than during follow-up. This finding is consistent with a study evaluating ventricular activation using electrocardiographic mapping, in which the baseline activation delay vector predicted CRT response, whereas CRT-induced changes in depolarization showed limited predictive value.^23^ Probably, after the initiation of pacing, the more important role in CRT response is played by spatial characteristics of ventricular repolarization rather than depolarization,^21^ since ventricular contraction takes place during repolarization phases of the action potential. Therefore, the sequence of depolarization appears to be a crucial component for predicting CRT response rather than for assessing the effects of CRT during the follow-up period.

## Limitations

Our study has demonstrated original findings and a novel approach in dyssynchrony evaluation, but some limitations are to be taken into account. (i) The computation of the QRS path directions was based on a generic heart/torso model derived from a 57-year-old male rather than patient-specific anatomy. Although some reduction in patient-specific accuracy is expected, the present approach, which evaluates depolarization gradients rather than detailed three-dimensional ventricular activation patterns, is considered sufficiently robust for the intended application and does not require CT or MRI imaging. (ii) A ≥10% reduction in LVESV was used to define response to CRT in our study, which differs from the ≥15% threshold applied in other studies and may limit direct comparability with those findings. (iii) The findings in the multivariate analysis (*Table 4*), based on five included variables and a relatively limited population, warrant further validation in independent cohorts.

## Conclusions

The CineECG-derived ventricular depolarization gradients reflecting the depolarization sequence in ventricular myocardium demonstrated significant changes after the CRT implant. The pre-implantation apicobasal depolarization gradient predicted the echocardiographic outcome of CRT independently of QRS duration. Specifically, the positive haemodynamic response was associated with the less pronounced apicobasal depolarization gradient.

## Data Availability

Data cannot be shared for ethical/privacy reasons.
